# Effect of Nickel and Cobalt on Methanogenic Enrichment Cultures and Role of Biogenic Sulfide in Metal Toxicity Attenuation

**DOI:** 10.3389/fmicb.2017.01341

**Published:** 2017-07-18

**Authors:** Lara M. Paulo, Javier Ramiro-Garcia, Simon van Mourik, Alfons J. M. Stams, Diana Z. Sousa

**Affiliations:** ^1^Laboratory of Microbiology, Wageningen University Wageningen, Netherlands; ^2^Farm Technology Group, Plant Sciences Group Wageningen, Netherlands; ^3^Centre of Biological Engineering, University of Minho, Campus de Gualtar Braga, Portugal

**Keywords:** heavy metals, anaerobic sludge, sulfate, sulfide, inhibition, stimulation

## Abstract

Metals play an important role in microbial metabolism by acting as cofactors for many enzymes. Supplementation of biological processes with metals may result in improved performance, but high metal concentrations are often toxic to microorganisms. In this work, methanogenic enrichment cultures growing on H_2_/CO_2_ or acetate were supplemented with trace concentrations of nickel (Ni) and cobalt (Co), but no significant increase in methane production was observed in most of the tested conditions. However, high concentrations of these metals were detrimental to methanogenic activity of the cultures. Cumulative methane production (after 6 days of incubation) from H_2_/CO_2_ was 40% lower in the presence of 8 mM of Ni or 30 mM of Co, compared to controls without metal supplementation. When acetate was used as substrate, cumulative methane production was also reduced: by 18% with 8 mM of Ni and by 53% with 30 mM of Co (after 6 days of incubation). Metal precipitation with sulfide was further tested as a possible method to alleviate metal toxicity. Anaerobic sludge was incubated with Co (30 mM) and Ni (8 mM) in the presence of sulfate or sulfide. The addition of sulfide helped to mitigate the toxic effect of the metals. Methane production from H_2_/CO_2_ was negatively affected in the presence of sulfate, possibly due to competition of hydrogenotrophic methanogens by sulfate-reducing bacteria. However, in the enrichment cultures growing on acetate, biogenically produced sulfide had a positive effect and more methane was produced in these incubations than in similar assays without sulfate addition. The outcome of competition between methanogens and sulfate-reducing bacteria is a determinant factor for the success of using biogenic sulfide as detoxification method.

## Introduction

Heavy metals are common pollutants in wastewaters, such as those from metal plating, mining, or pulp and paper industries (Sarioglu et al., 2010). Heavy metals may have a dual effect on microorganisms present in anaerobic wastewater treatment systems. Metals have a fundamental role in structural or catalytic functions in cells and can be beneficial to certain biological processes (Ehrlich, 1997). On the other hand, high metal concentrations are often toxic to microorganisms (Chen et al., 2008).

Positive effects of metal supplementation on the anaerobic digestion of wastewaters have been reported, especially for Ni and Co (Gustavsson et al., 2011, 2013; Karlsson et al., 2012). This is explained by the importance of these metals in enzymes that are involved in methanogenesis; Ni is present in Ni-Fe hydrogenases and in the cofactor F_430_ (the prosthetic group of methyl coenzyme M reductase), while Co is present in cobalamides (DiMarco et al., 1990). Cobalamides have an important role as methyl carriers in methanogenesis from methylated compounds as they are intermediates between methyl-H_4_MPT and coenzyme M (DiMarco et al., 1990). Moreover, methyl-H_4_MPT:CoM-SH methyltransferase has in one of its subunits a cob(I)amide prosthetic group that is believed to be important for the enzymatic function (Hedderich and Whitman, 2006).

Recently, Chen et al. (2016) observed the stimulation of anaerobic digestion by Co and Ni up to concentrations of 1.7 mM, above which toxic effects started to be noticeable. Distinct toxic concentrations of these metals have been reported in other studies, possibly due to the large variation in experimental setups used and of inocula tested (Chen et al., 2008; Paulo et al., 2015). For example, toxic effects of Ni have been observed in a range of concentrations above ∼ 0.5–50 mM, depending on conditions (Paulo et al., 2015). Co toxicity has been less reported in literature, but a reference value is in the order of 15 mM (Bhattacharya et al., 1995). The toxic effect of heavy metals can be due to their ability to disrupt enzyme functions and structures by binding with thiol and other groups in proteins (Chen et al., 2008; Colussi et al., 2009). Co can inactivate Fe-S proteins, interfere with sulfur metabolism and Fe-S cluster biogenesis, and cause the formation of reactive oxygen species (Eitinger, 2013). As for Ni, it can replace Fe in many enzymes or bind to cysteine, histidine or negatively charged residues in active sites of non-metal enzymes (Eitinger, 2013). Microorganisms have developed different strategies for metal resistance, including metal efflux proteins, excretion of chelating agents, biomethylation, or ion reduction (Paulo et al., 2015). Although these mechanisms are widely studied in bacteria, in methanogens they are not yet well characterized (Maezato and Blum, 2012). Nevertheless, three of the six identified Ni/Co transporters systems used for metal efflux can be found in methanogens: the Nik/CbiMNQO, NikABCDE, and NiCoT (Bini, 2010). One of the NikABCDE components, the NikA, is suspected to be involved in Ni sequestration in case of metal excess conditions (Eitinger and Mandrand-Berthelot, 2000). Putative homologs of the *rcnA* gene, known to codify a nickel defense system, were also detected in archaea (Macomber and Hausinger, 2011). The expression of *rcnA* is regulated by RcnR, a nickel/cobalt responsive repressor (Macomber and Hausinger, 2011). CznABC efflux pumps are members of the RND family and found in archaea groups as well (Eitinger, 2013). In *Helicobacter* species these pumps have been associated with the export of Cd, Zn, and Ni (Sawers, 2013).

A proposed method to decrease dissolved metal concentrations in wastewaters and, in this way attenuate their toxicity toward microorganisms, is the precipitation with sulfide. In anaerobic digestion sulfide is biogenically formed from the degradation of S-containing organic compounds or by the action of sulfate-reducing bacteria (SRB). Sulfide reacts quickly with several metal ions forming insoluble metal-sulfides (Zayed and Winter, 2000). Despite its proven effectiveness to precipitate metals, the effects of using biologically produced sulfide on methane production or on the composition of microbial communities are not extensively studied.

In this study, low and high concentrations of Ni and Co were added to anaerobic sludge and the potential stimulatory and toxic effects of these metals on hydrogenotrophic and aceticlastic activities were evaluated. High metal levels were detrimental for methane production and, for this reason, a detoxification strategy involving the precipitation of metals with sulfide was further on studied. The aim of this work was to study possible approaches to maximize methane production in wastewaters containing heavy metals.

## Materials and Methods

### Inoculum Sludge, Medium Composition, and Culture Conditions

Granular anaerobic sludge was obtained from a wastewater treatment plant treating food industry effluent (Delft, The Netherlands). The sludge was washed, and about 2 g of volatile suspended solids (VSS) were used to inoculate vials containing 50 mL of basal medium. Medium was prepared according to the protocol previously described by Stams et al. (1993), with the following exceptions: no NaHCO_3_ was added and buffering of the medium was done with 20 mM HEPES [4-(2-hydroxyethyl)-1-piperazineethanesulfonic acid] (pH was corrected to 7.0); titanium citrate (0.2–0.3 mM) was amended as reducing agent instead of Na_2_S (to avoid metal precipitation). HEPES was chosen as buffer because it does not react with metal ions (Ferreira et al., 2015). The effect of metals on aceticlastic and hydrogenotrophic activities was tested in separate assays. Aceticlastic activity was measured in the presence of 20 mM of sodium acetate and using N_2_ as the headspace gas (1.5 atm). Hydrogenotrophic activity was measured using as sole substrate 1.5 atm of H_2_/CO_2_ (80:20; % v/v). Metals, sulfate and sulfide were added to assay vials from anaerobic stock solutions to the final desired concentrations, as detailed in the next section. All the materials (bottles, rubber stoppers) were previously washed with 3 M nitric acid to avoid any contamination with other metals. All solutions were prepared with ultrapure water. The assays were performed in the dark at 30°C and stirred at 100 rpm.

### Metal, Sulfate, and Sulfide Supplementation Tests

Effect of Ni and Co was assayed at low (supplementation test, Ni-L and Co-L) and high (toxicity test, Ni-H and Co-H) concentrations. Final concentrations of NiCl_2_ were 2, 4, and 8 μM (Ni-L) and 2, 4, and 8 mM (Ni-H). For Co tests, CoCl_2_ was added to final concentrations of 5.5, 10.5, and 25.5 μM (Co-L) and 2.5, 5, 10, 20, and 30 mM (Co-H). These final concentrations are corrected for the Ni and Co already present in the basal medium. Controls without metal addition were included. Sulfate, as Na_2_SO_4_, was added to the bottles with high metal concentrations; different metal to sulfate ratios were tested (i.e., 0.5:1; 1:1; 1.5:1, mol:mol). Moreover, bottles with Na_2_S plus metals were also prepared (ratios 0.5:1; 1:1; 1.5:1, mol:mol). pH of Na_2_S stock solution was corrected to 7.0. All conditions were tested in duplicate. A table with all the different conditions and codes for all the assays is included in Supplementary Table S1. Methane production as well as fatty acid, sulfate and sulfide concentrations were monitored over a 6-days incubation period.

### Analytical Methods

Gas in bottles’ headspace was sampled using a gas tight syringe and analyzed for H_2_ and CH_4_ with a Compact GC^4.0^ (Global Analyser Solutions, Breda, Netherlands) equipped with Carbonex 1010 column (Supelco, 3 m × 0.32 mm) followed by a Mosieve 5A column (Restek, 30 m × 0.32 mm) and a thermal conductivity detector (TCD). Argon was used as carrier gas at 0.8 mL min^-1^. Standard GC settings for H_2_ and CH_4_ measurement were: 300 kPa; valve (injection) oven: 60°C; column oven: 100°C; TCD temperature: 100°C; filament: 175°C.

Liquid samples were analyzed for volatile fatty acids (VFA) and alcohols with an Accela HPLC (Thermo Scientific, Waltham, MA, United States) equipped with a Varian Metacarb 67H column (Agilent, 300 mm × 6.5 mm) and a refractive index detector. Column was kept at 45°C and running with 0.01N of H_2_SO_4_ as eluent at a flowrate of 0.8 mL/min.

Sulfate concentrations were measured in an ICS-2100 Ion-Chromatograph system (Thermo Scientific) equipped with an AS19 column (250 mm × 2 mm) using a hydroxide (gradient) solution as eluent. Sulfide was measured using the methylene blue method, as described by Trüper and Schlegel (1964).

### Microbial Community Analysis

At the end of the assays, 2 mL aliquots were withdrawn from the vials and frozen at -80°C for further DNA extraction. DNA was extracted using the FastDNA^TM^ Spin Kit for Soil DNA Extraction (MPBio, Santa Ana, CA, United States). After purification, DNA was amplified and representative samples of each tested condition were chosen for sequencing in Miseq platform. Barcoded amplicons targeting the V4 region of the 16S rRNA were generated using a 2-step PCR strategy in order to reduce the impact of barcoded primer on the outcome of the microbial profiling (Berry et al., 2011). The 10–20 ng of DNA was used as template in the first PCR reaction (50 μL) which contained 10 μL HF buffer (Finnzymes, Vantaa, Finland), 1 μL dNTP Mix (10 mM; Promega, Leiden, Netherlands), 1 U of Phusion^®^ Hot Start II High-Fidelity DNA polymerase (Finnzymes), 500 nM of each primer (UniTag1-515f (GAGCCGTAGCCAGTCTGC-GTGYCAGCMGCCGCGGTAA) and UniTag2-806r (GCCGTGACCGTGACATCG-GGACTACNVGGGTWTCTAAT)(Walters et al., 2016). The selection procedure of the UniTags is described elsewhere (Tian et al., 2016). PCRs were performed with a SensoQuest Labcycler (Göttingen, Germany) using an adaptation of the cycling conditions of Caporaso et al. (2012). The cycling conditions for the first step consisted of an initial denaturation at 98°C for 3 min, 25 cycles of: 98°C for 10 s, 50°C for 20 s, and 72°C for 20 s, and a final extension at 72°C for 10 min. The size of the PCR products (∼330 bp) was confirmed by agarose gel electrophoresis using 5 μL of the amplification-reaction mixture on a 1% (w/v) agarose gel containing 1× SYBR^®^ Safe (Invitrogen, Carlsbad, CA, United States).

Five microliters of the first PCR reaction were used as template for the second PCR reaction (100 μL), which contained 20 μL HF buffer, 2 μL dNTP Mix, 2 U of Phusion^®^ Hot Start II High-Fidelity DNA polymerase and 500 nM of UniTag1 (forward) and Unitag2 (reverse), each. UniTag1 and Unitag 2 were appended with an 8 nt sample specific barcode at the 5′end (Ramiro-Garcia et al., 2016). The cycling conditions consisted of an initial denaturation at 98°C for 30 s, followed by five cycles of: 98°C for 10 s, 52°C for 20 s, and 72°C for 20 s. A final extension at 72°C for 10 min followed the cycles. The incorporation of the specific barcodes (PCR product of ∼350 bp) was confirmed by gel electrophoresis.

The final PCR products were purified with HighPrep^TM^ (Magbio Genomics, Gaithersburg, MD, United States) using 20 μL of Nuclease Free Water (Promega, Madison, WI, United States) for elution and quantified using a Qubit in combination with the dsDNA BR Assay Kit (Invitrogen, Carlsbad, CA, United States). The purified products were mixed together in equimolar amounts to create a library pool and sent for sequencing on the Miseq platform (GATC Biotech AG, Konstanz, Germany). Data was analyzed using NG-Tax, a validated pipeline for 16S rRNA analysis (Ramiro-Garcia et al., 2016). Sequence data have been deposited in the European Nucleotide Archive, accession number [ENA: PRJEB20620].

### Data Analysis

A modified Gompertz model (Eq. 1) was used to fit the data from CH_4_ measurements:

(1) $$M(t)=P\;\text{exp}\left[-\exp\left[\frac{R_{\text{m}}e}{P}(\lambda -t)+1\right]\right]$$

in which, *M* (mmol/L) is the cumulative methane production, *P* (mmol/L) is the methane production potential, *R*_m_ (mmol/L.h^-1^) is the rate of production, λ (h) is the lag phase time, *e* is exp (1), and t (h) is time (Lay et al., 1997). An example of data fit is given in the Supplementary Figure S1.

For statistical comparison of different conditions, the probability distribution of the parameter vector [*P R*_m_ λ] was obtained by a Markov chain Monte Carlo sampling method (ter Braak and Vrugt, 2008), and summarized by its 95% confidence region, following the methodology described by van Mourik et al. (2014). Sampling was carried out in log-space, i.e., after a log transformation of the parameter vector. For estimating the maximum likelihood of the parameter vector we used a hybrid algorithm consisting of a global search of the genetic search routine ’GA’ in Matlab^®^ software with a population of 200 with a maximum of 10^3^ generations, followed by a gradient based search with the ‘lsqnonlin’ routine in Matlab^®^, starting from the optimum found by ‘GA.’ For Monte Carlo sampling, we used three chains, 2 × 10^4^ iterations per chain, 2 × 10^3^ burn-in iterations, and a thinning rate of 10. We used a log-uniform prior. The confidence regions were represented with 300 samples.

An example of the 95% confidence curves obtained for two of the studied conditions (Control and Ni-H-8, hydrogenotrophic) is given in Supplementary Figure S2. Three time points (*t*_20_, *t*_60_, *t*_120_, time in hours) were selected for comparison between different conditions. These time points are representative of different growth phases: the two first time points allow to compare differences in lag phase (when present) and initial methane production, while the last point represents the final cumulative methane production.

### Genomic Searches

An analysis of the genomes of *Methanobacterium formicicum* JCM 10132 (Taxonomy ID: 1300163), *Methanosaeta concilii* GP6 (Taxonomy ID: 990316), *Methanosarcina barkeri* CM1 (Taxonomy ID: 796385) and *Methanolinea* sp. SDB (Taxonomy ID: 1735327) was performed using the online tool Rast^1^ (Aziz et al., 2008; Overbeek et al., 2014; Brettin et al., 2015) and checked the presence of genes related with Ni/Co transport and metal resistance in these microorganisms. RcnA could not be found in Rast search for resistance subsystems. For this reason, RcnA protein sequence (NC-000913.3) (Rodrigue et al., 2005) was also searched using the online tool BLAST^2^ and compared with the genomes of the methanogens. These methanogens were chosen as they represent the abundant archaeal genera present in our samples. *Methanosarcina barkeri*, was not abundant in our sludge, but it was included in the analysis because this is the best studied methanogen in what concerns metal toxicity. *M. barkeri* is often present in anaerobic sludges (Ziganshin et al., 2013; Venkiteshwaran et al., 2015).

## Results

### Metal Supplementation and Toxicity Tests

**FIGURE 1:**
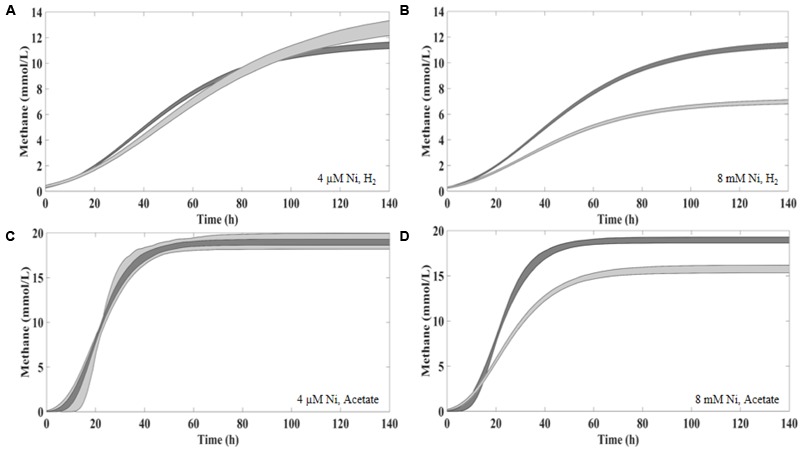
The 95% confidence curves between the Control and 4 μM of Ni **(A,C)** and 8 mM of Ni **(B,D)** supplementation in the presence of H_2_/CO_2_ **(A,B)** and acetate **(C,D)**. Control curves in dark gray.

Cumulative methane production values measured after 20, 60, and 120 h of incubation of the anaerobic sludge are shown in **Table 1** (incubations with Ni) and **Table 2** (incubations with Co). These values were obtained from the methane production curves, with deviations calculated for confidence level of 95%. Examples of methane production curves from both stimulation and toxicity assays are shown in **Figure 1**. In the assays with H_2_/CO_2_ as substrate, no VFA were detected in the medium (data not shown). Regarding the test with acetate, no other VFAs were produced and acetate consumption could be stoichiometrically correlated to the formation of methane (data not shown).

**Table 1:** Cumulative methane production measured (mmol L^-1^) at three different time points for each condition in the presence of Ni.

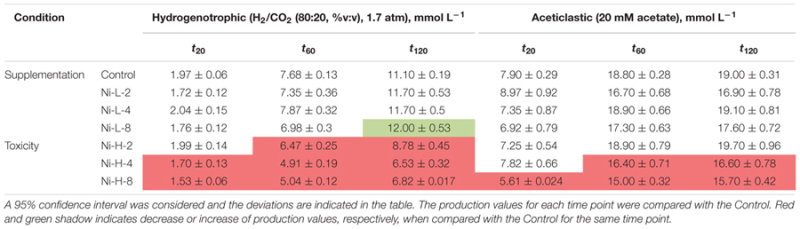

**Table 2:** Cumulative methane production measured (mmol L^-1^) at three different time points for each condition in the presence of Co.

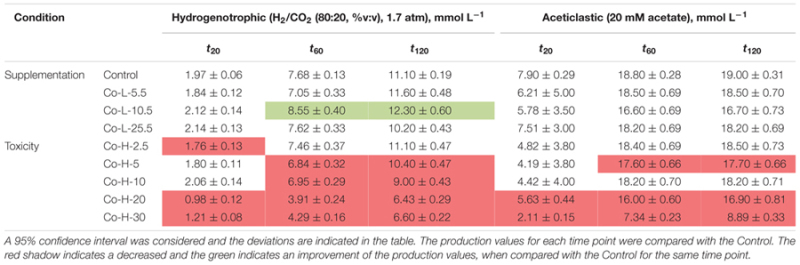

Supplementation with 8 μM Ni resulted in approximately 10% higher cumulative methane production from H_2_/CO_2_ (at *t*_120_, compared to the control), but the same effect was not observed for lower amounts of Ni (i.e., 2 and 4 μM Ni). On the other hand, high concentrations of Ni (i.e., 2, 4, and 8 mM) had a detrimental effect on methanogenesis from H_2_/CO_2._ When compared with the controls, cumulative methane production (*t*_120_) decreased by approximately 20% when 2 mM of Ni were added and by 40% with 4 and 8 mM of Ni. Aceticlastic methanogenesis did not benefit from Ni supplementation, and was negatively affected by high Ni concentrations (decrease of 11 and 18% on the final cumulative methane production at concentrations of 4 and 8 mM of Ni, respectively).

Supplementation of 10.5 μM of Co to hydrogen-consuming cultures was beneficial, but also for this metal, high Co concentrations (higher than 5 mM) had a negative effect (**Table 2**)_._ Aceticlastic methanogenesis was not affected by Co supplementation, but the toxic effect of Co was noticeable for concentrations ≥20 mM; cumulative methane production (*t*_120_) decreased by ∼11% and ∼53% for concentrations of 20 and 30 mM Co respectively.

### Sulfide as Metal Detoxification Method

**FIGURE 2:**
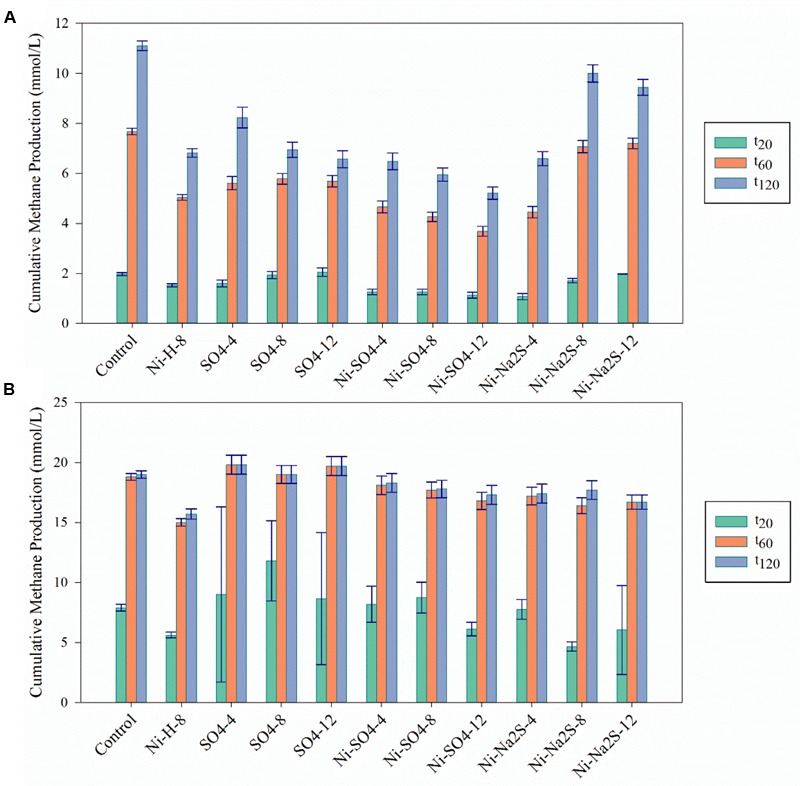
Cumulative methane production for the Ni detoxification assays at the three different time points (*t*_20_; *t*_60_; *t*_120_) in **(A)** in the presence of H_2_/CO_2_ and **(B)** in the presence of acetate. The black bars indicate the 95% confidence intervals. H/L – high/low metal concentration (mM/mM orders); numbers indicate concentrations of metals and sulfate/sulfide (Supplementary Table S1 for detailed description).

**FIGURE 3:**
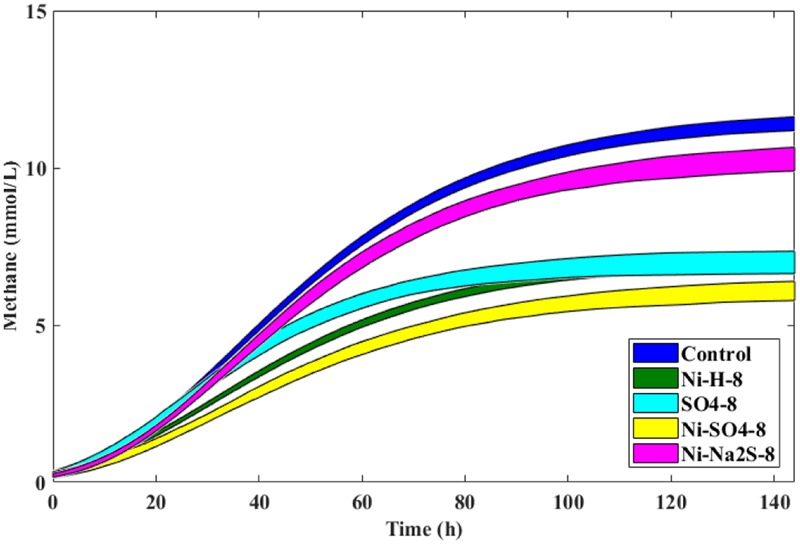
The 95% confidence regions for the different conditions in the presence of H_2_/CO_2_ and 8 mM of Ni, with and without sulfate/sulfide, and control. Sulfate/Sulfide were added to a final concentration of 8 mM.

**FIGURE 4:**
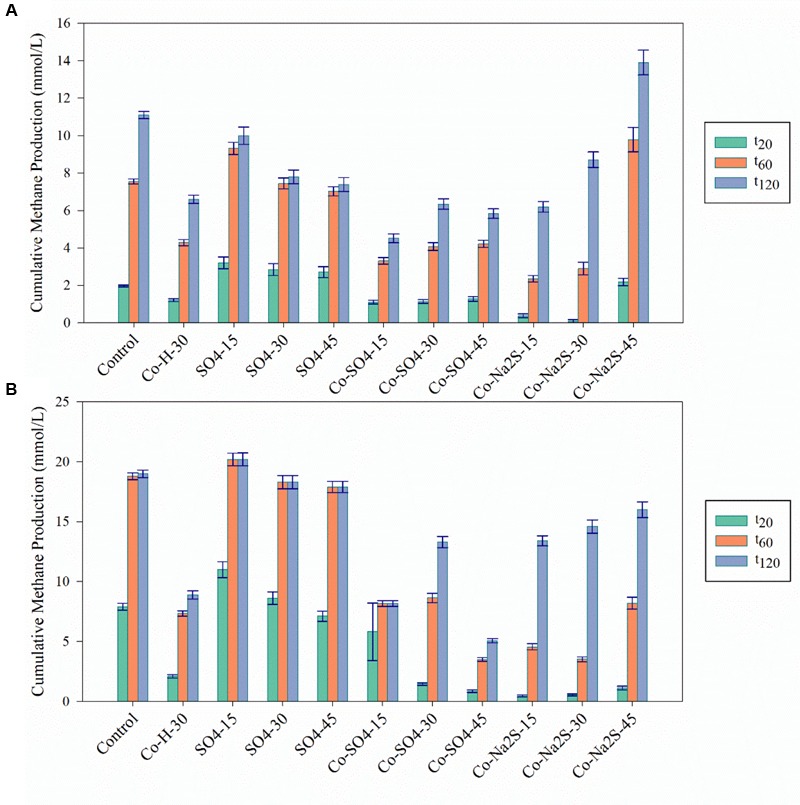
Cumulative methane production values for the Co detoxification assays at the three different time points (*t*_20_; *t*_60_; *t*_120_) in **(A)** in the presence of H_2_/CO_2_ and **(B)** in the presence of acetate. The black bars indicate the 95% confidence intervals. H stands for High concentration, L for Low and M for Moderate. H/L – high/low metal concentration (mM/mM orders); numbers indicate concentrations of metals and sulfate/sulfide (Supplementary Table S1 for detailed description).

To evaluate the potential of sulfide (produced by SRB) as metal detoxification agent, anaerobic sludge was incubated with 8 mM of Ni or 30 mM of Co plus sulfate. Incubations with sulfate (no metal) and with sulfide (Na_2_S) plus metal were also performed as controls. The results are depicted in **Figures 2–4** and **Table 3**.

**Table 3:** Percentage of Na_2_SO_4_ consumption in assays with Na_2_SO_4_, in the absence and presence of Ni or Co.

	% of SO_4_ consumption	
Condition/Substrate	H_2_/CO_2_	Acetate
SO_4_-4	97.01	44.22
Ni-SO_4_-4	7.91	18.34
SO_4_-8	76.86	35.49
Ni-SO_4_-8	2.04	23.19
SO_4_-12	64.21	34.54
Ni-SO_4_-12	7.48	20.15
SO_4_-15	46.62	25.57
Co-SO_4_-15	33.14	26.97
SO_4_-30	43.7	26.64
Co-SO_4_-30	31.77	0
SO_4_-45	10.7	5.44
Co-SO_4_-45	0.74	0

**FIGURE 5:**
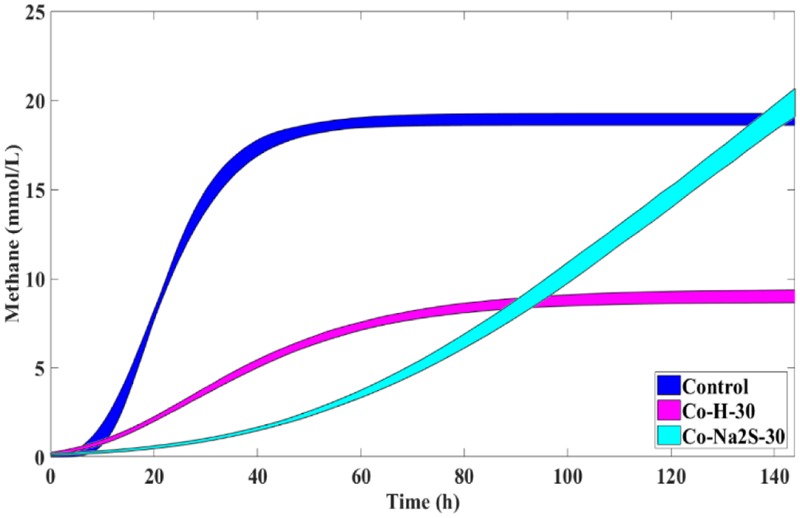
Cumulative methane production curves (with uncertainty) obtained for H_2_/CO_2_ incubations with 30 mM of Co with and without Na_2_S and the respective control without metal addition (nor sulfate or sulfide).

The addition of sulfate together with 8 mM of Ni or 30 mM of Co had no beneficial effect on methane production from H_2_/CO_2_. However, direct addition of Na_2_S to assays with either Ni or Co helped to alleviated metal toxicity in most of the cases (both methane production rate and final cumulative methane concentration). This is illustrated in **Figure 3**, where we show methane production profiles from H_2_/CO_2_ in the presence of Ni, with and without sulfate or Na_2_S. For Co, the effect of adding sulfide on metal toxicity mitigation is not immediate and initial rates of H_2_/CO_2_ conversion to methane are still in effect, although this addition has an effect on the final recovery of methane (**Figure 5**). For aceticlastic cultures, the addition of 15 mM to 30 mM of sulfate together with Co had beneficial effects on methane production rate and/or cumulative maximum methane production (**Figure 4B**). However, when assays where amended with 45 mM sulfate, methane production was even lower than in the presence of only the metal.

### Microbial Community Analysis

**FIGURE 6:**
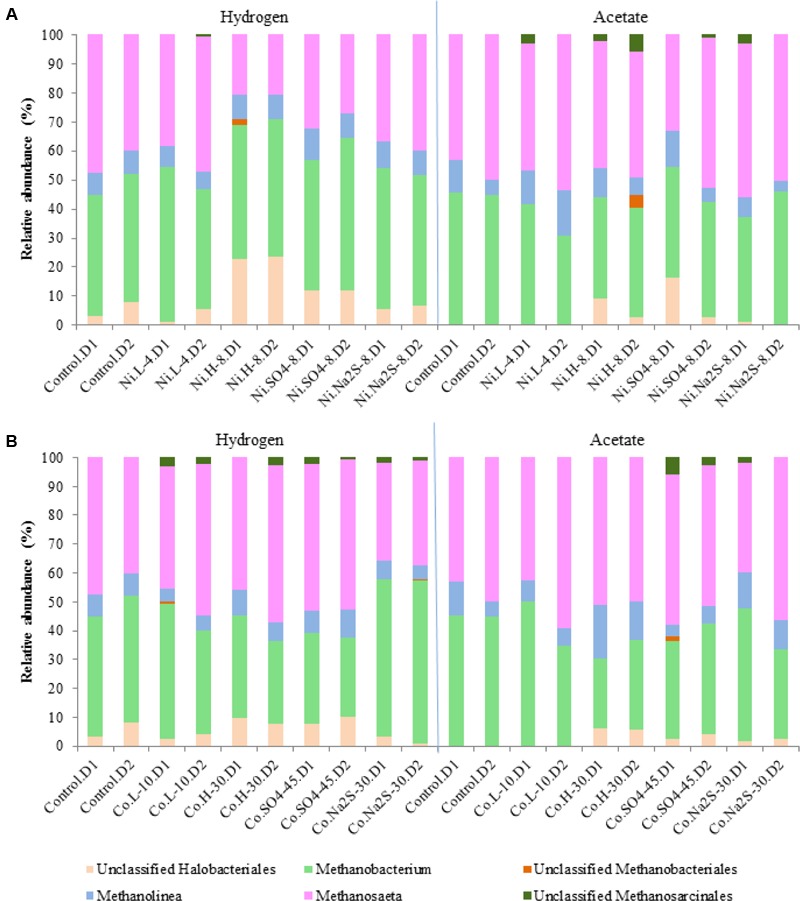
Relative abundances for Archaeal diversity (genus level) in the presence of Ni **(A)** and Co **(B)**. D1 and D2 refers to the biological replicate 1 and 2 of each condition.

The archaeal community (**Figure 6**) was dominated by species of *Methanobacterium* (hydrogenotrophic methanogens) and *Methanosaeta* (aceticlastic methanogens), which together represented 70 to 96% of the total archaeal community in the samples. *Methanolinea*, another genus of hydrogenotrophic methanogens, represented about 4-16% of the archaeal community. Unclassified species of *Halobacteriales* (1–23%) were also detected in most of the samples. In the presence of 8 mM of Ni, with H_2_/CO_2_ as substrate, there was a change in the relative abundances of *Methanosaeta* from 40–48% to ∼21%, while *Halobacteriales* relative abundances increased to 20%. Relative abundances of *Methanobacterium* and *Methanolinea* members in H_2_/CO_2_-grown cultures showed minute variations in the presence of Ni (all range of tested concentrations). However, in the presence of 30 mM Co *Methanobacterium* decreased from 42–46% to 28–35%. As expected, when acetate was the supplied substrate, members of *Methanobacterium* decreased from ∼45% to 24–31% in the presence of the metals. *Halobacteriales* were absent in the assays growing with acetate, without or with low concentrations of metals, while they appeared in samples with metal plus sulfate or sulfide in relative abundances up to 16%.

**FIGURE 7:**
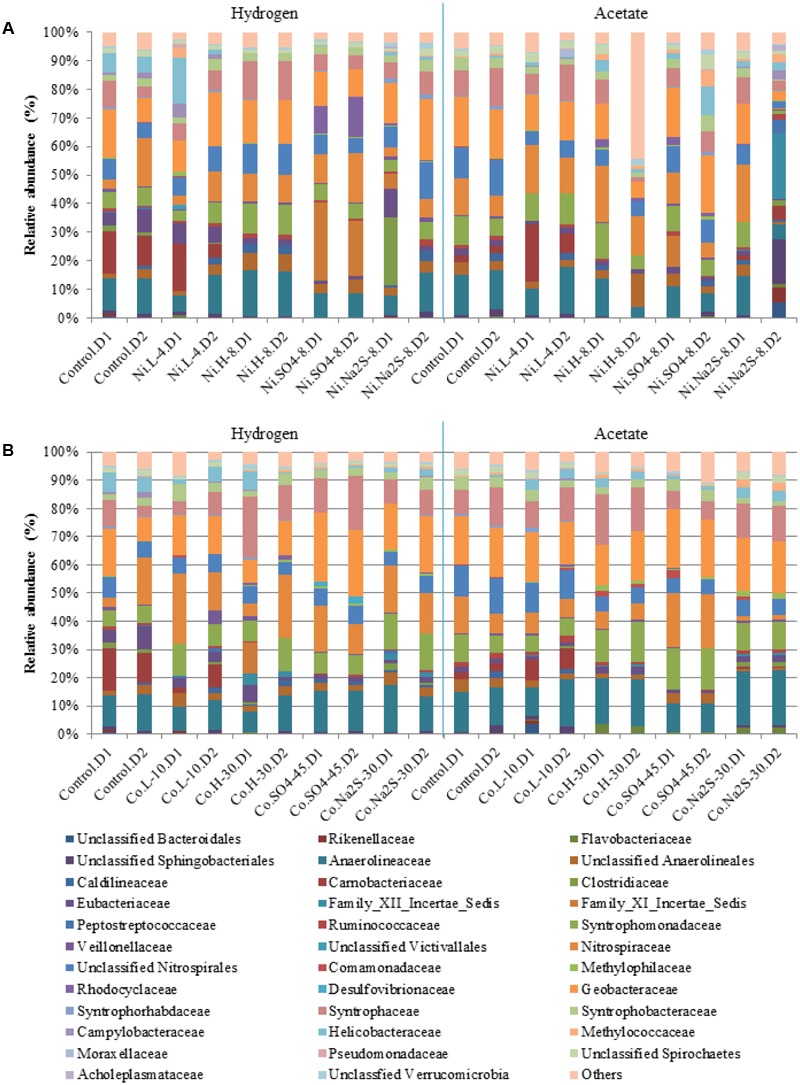
Relative abundances for Bacterial diversity (family level) in the presence of Ni **(A)** and Co **(B)**. D1 and D2 refers to the biological replicate 1 and 2 of each condition.

Bacterial communities were dominated by *Anaerolineaceae* (3 to 13%), *Syntrophomonadaceae* (3 to 12.5%), *Nitrospiraceae* (2 to 18%), *Geobacteraceae* (5 to 19%), and *Syntrophaceae* (1.7 to 17.5%), while other families were present in smaller abundances (**Figure 7**). *Carnobacteriaceae* members, which in hydrogenotrophic samples represented up to 13% of the community, were not present in samples containing high concentrations of Co, Ni, sulfate, or sulfide. Relative abundances of *Eubacteriaceae*, which were more dominant in hydrogenotrophic incubations (up to 7%), also changed in the presence of metal, sulfate, or sulfide. *Rhodocyclaceae* members, specifically of the genus *Azospira*, were present in the hydrogenotrophic samples containing Ni and sulfate, representing 8 to 12% of the community in these samples. *Desulfovibrio* species could also be detected, but only in incubations with 45 mM of sulfate and Ni.

### Analysis of the Genomes of *M. formicicum, Methanolinea* sp., *M. barkeri*, and *M. concilii*

Genes encoding for CbiMNQO and NikMQO transport systems could be found in the genomes of *M. formicicum* JCM, *M. concilii* GP6, *M. barkeri* CM1, and *Methanolinea* sp. SDB. Furthermore, *M. barkeri* genome contains genes encoding for the NikABCDE system, including the NikA subunit. *M. concilii, M. formicium*, and *M. barkeri* possess genes encoding for NikN. In addition, all four genomes have multiple homologs encoding for CzcD, which is a known cobalt-zinc-cadmium resistance protein. The Ni and Co resistance gene from *E. coli* RcnA was also searched in the genomes of the methanogens but no significant hits were retrieved.

## Discussion

Ni and Co are important metal ions for methanogenesis and their supplementation to anaerobic bioreactors may be beneficial for methane production (Gustavsson et al., 2011; Evranos and Demirel, 2015). However, several studies show that no significant enhancement is obtained from metal addition, or that the benefit is dependent on the substrate fed to the microorganisms (Zandvoort et al., 2006b; Park et al., 2010). In the present work, supplementation of Ni and Co to anaerobic sludge converting H_2_/CO_2_ or acetate resulted in little or no increase of methane production. One explanation for this could be that metals adsorbed to the anaerobic sludge are sufficient to fulfill the nutritional requirements of the microorganisms. Metal adsorption in biofilms (including granules) is a known phenomenon (van Hullebusch et al., 2003, 2004, 2005; Zandvoort et al., 2006a). It has been shown that the supplementation of Co and Ni in systems that were metal deprived helps in restoring methane production and full substrate degradation (Fermoso et al., 2008a,b, 2010).

Heavy metals can accumulate in anaerobic bioreactors to toxic concentrations for microorganisms. In this study, hydrogenotrophic activity was more affected in the presence of Ni than aceticlastic activity. For example, 8 mM of Ni caused a decrease in final methane production from H_2_/CO_2_ by ∼40% and only by 18% from acetate. However, no relevant differences in the relative abundances of hydrogenotrophic and aceticlastic methanogens were observed in assays with or without Ni. *Methanosaeta* or *Methanolinea* species were the predominant aceticlastic methanogens in the anaerobic sludge, and their resistance to Ni has never been assessed. *Methanobacterium* species are reported to be quite resistant to Ni; in a pure culture study *M. formicium* was shown to be more resistant to Ni than *M. barkeri* or *Methanothermobacter marburgensis* (Jarrell et al., 1987). Similarly, it was reported that *M. formicicum* was not affected by Ni concentrations up to 1200 mg/L (∼20 mM), while *Methanosarcina thermophila* was inhibited with 500 mg/L of Ni (∼8.5 mM) (Sanchez et al., 1996). Considering Co, the results indicate a higher toxicity threshold (20 and 30 mM), both for hydrogenotrophic and aceticlastic methanogens. An apparent decrease in the relative abundance of *Methanobacterium* species was observed in the presence of Co, and this result is corroborated by the decrease in methane production observed from H_2_/CO_2_.

Our results show a clear effect of high concentrations of Ni and Co on methanogenesis but, in most cases, this could not be linked with variations in the microbial composition. The fact that we used a DNA-based analysis, and considering the short incubation times (∼150 h), could be a reason for the lack of visible changes in microbial communities. The doubling time of some methanogenic species, such as *Methanolinea mesophila* and most *Methanobacterium* species^3^ (online August 2016) (Jablonski et al., 2015), is lower than 29 h under optimal conditions, while for other species, such as *Methanosaeta concilii*, the doubling times are of about 65 h (35°C, pH 7.8). Based on this, even a DNA-based analysis is expected to show some differences when there is microbial inhibition, which suggests that some of the methanogenic species present in the sludge could endure the presence of metals. Different mechanisms might be involved in metal resistance in microorganisms. The active efflux of Ni and Co is likely the detoxification mechanism used by most microorganism (Nies, 1992). A search of the genomes of *M. barkeri, M. concilii, Methanolinea* sp., and *M. formicium* revealed the presence of genes codifying for Ni and Co transport systems, including CzcD system. Metal resistance mechanisms and its regulation systems in archaea are still not well understood yet.

Precipitation of metals with sulfide is a potential metal detoxification method, but the competition between SRB and methanogens for common substrates needs to be taken into account. In the case of hydrogenotrophic activity, the addition of sulfate to cultures with high metal concentration had little beneficial effect on methane production, while the addition of Na_2_S helped to restore the methane production. This is possibly explained by the strong competition between methanogens and SRB for hydrogen. Not only the reduction of sulfate from hydrogen is thermodynamically more favorable than methane production, but it is also known that SRB have a higher affinity for hydrogen than methanogens and, for that, SRB are expected to outcompete methanogens (Colleran et al., 1995). Moreover, the metal can also be toxic for the SRB present in the sludge and inhibit their performance, which can be seen by the decrease of sulfate consumption in the presence of metal. Studies performed in pure cultures of *Desulfovibrio desulfuricans* showed that this microorganism is sensitive to Ni concentrations above 0.17 mM (Poulson et al., 1997). *Desulfovibrio vulgaris* and *Desulfovibrio* sp. cultures were severely inhibited by similar Ni concentrations (0.14 mM) (Cabrera et al., 2006). Other SRB species can tolerate higher concentrations, as for example *Desulfotomaculum* sp. that can tolerate 9.5 mM of Ni (Fortin et al., 1994). In our study, the high concentrations of Ni tested (2, 4, and 8 mM) were above the toxic concentrations reported to *Desulfovibrio* species and we observed very low sulfate removal percentages in the presence of metal. The presence of sulfide (biogenically produced or Na_2_S) in high Ni cultures with hydrogen resulted in higher numbers of *Methanosaeta*. This indicates that the presence of sulfide can help to diminish the toxic effects of metals for some microorganisms and that metal toxicity can be reversed. In our assays with sulfate and acetate, no decrease in methane production was observed. Moreover, sulfate consumption in the assays using acetate as substrate was relatively small, especially for high sulfate concentrations (less than 25%). The addition of sulfate together with high metal concentration had some beneficial effect as metal detoxification method, but the values of cumulative methane production were lower than when sulfate alone was present. Addition of Na_2_S had a positive effect in restoring methane production by the sludge, which shows the potential of metal toxicity attenuation by precipitation with sulfide.

## Conclusion

Addition of low concentrations of Ni and Co did not stimulate methanogenesis. It is possible that this effect is only observable in long-term incubation, or enrichment procedures, when residual metals reach limiting levels. Effect of metal addition is variable, depending on inoculum and environmental conditions, the range of metals and conditions tested should be amplified and the differences between methanogenic metabolic routes considered. Sulfide can be an efficient method of metal detoxification, but when considering biogenically produced sulfide competition between SRB and methanogens is a key factor that limits the efficiency of the method.

## Author Contributions

LP, AS, and DS designed the study; LP performed the experiments, analyzed the data, and wrote the manuscript; JR-G analyzed the sequencing data; SvM performed statistical analysis of data; DS and AS critically revised the manuscript. All authors read and approved the final manuscript.

## Conflict of Interest Statement

The authors declare that the research was conducted in the absence of any commercial or financial relationships that could be construed as a potential conflict of interest.

## Footnotes

1 http://rast.nmpdr.org/

2 https://www.ncbi.nlm.nih.gov/blast/

3 http://metanogen.biotech.uni.wroc.pl/
