# Supplementary material for: Effect of Nickel and Cobalt on Methanogenic Enrichment Cultures and Role of Biogenic Sulfide in Metal Toxicity Attenuation
Source: Front Microbiol. 2017 Jul 18;8:1341. doi: 10.3389/fmicb.2017.01341 (PMC5513950; doi:10.3389/fmicb.2017.01341)
Supplement: Supplementary file 1 [file Data_Sheet_1.docx]

**Supplementary Material**

**Table 1** – Different test conditions studied in this work (metal, sulphate and sulphide concentrations). In the last column, samples subjected to 16S rRNA gene sequencing are indicated.

| Short name | Metal conc. | Sulphate conc. | Sulphide conc. | Sample sent to sequencing |
| --- | --- | --- | --- | --- |
| No | - | - | - | X |
| Ni-L-2 | 2 µM Ni | - | - | - |
| Ni-L-4 | 4 µM Ni | - | - | X |
| Ni-L-8 | 8 µM Ni | - | - | - |
| Co-L-5 | 5 µM Co | - | - | - |
| Co-L-10 | 10 µM Co | - | - | X |
| Co-L-25 | 25 µM Co | - | - | - |
| Ni-H-2 | 2 mM Ni | - | - | - |
| Ni-H-4 | 4 mM Ni | - | - | - |
| Ni-H-8 | 8 mM Ni | - | - | X |
| Co-H-2.5 | 2.5 mM Co | - | - | - |
| Co-H-5 | 5 mM Co | - | - | - |
| Co-H-10 | 10 mM Co | - | - | - |
| Co-H-20 | 20 mM Co | - | - | - |
| Co-H-30 | 30 mM Co | - | - | X |
| SO_4_-4 | - | 4 mM | - | - |
| SO_4_-8 | - | 8 mM | - | - |
| SO_4_-12 | - | 12 mM | - | - |
| SO_4_-15 | - | 15 mM | - | - |
| SO_4_-30 | - | 30 mM | - | - |
| SO_4_-45 | - | 45 mM | - | - |
| Ni-SO_4_-4 | 8 mM Ni | 4 mM | - | - |
| Ni-SO_4_-8 | 8 mM Ni | 8 mM | - | X |
| Ni-SO_4_-12 | 8 mM Ni | 12 mM | - | - |
| Ni-Na_2_S-4 | 8 mM Ni | - | 4 mM | - |
| Ni-Na_2_S-8 | 8 mM Ni | - | 8 mM | X |
| Ni-Na_2_S-12 | 8 mM Ni | - | 12 mM | - |
| Co-SO_4_-15 | 30 mM Co | 15 mM | - | - |
| Co-SO_4_-30 | 30 mM Co | 30 mM | - | - |
| Co-SO_4_-45 | 30 mM Co | 45 mM | - | X |
| Co-Na_2_S-15 | 30 mM Co | - | 15 mM | - |
| Co-Na_2_S-30 | 30 mM Co | - | 30 mM | X |
| Co-Na_2_S-45 | 30 mM Co | - | 45 mM | - |

**
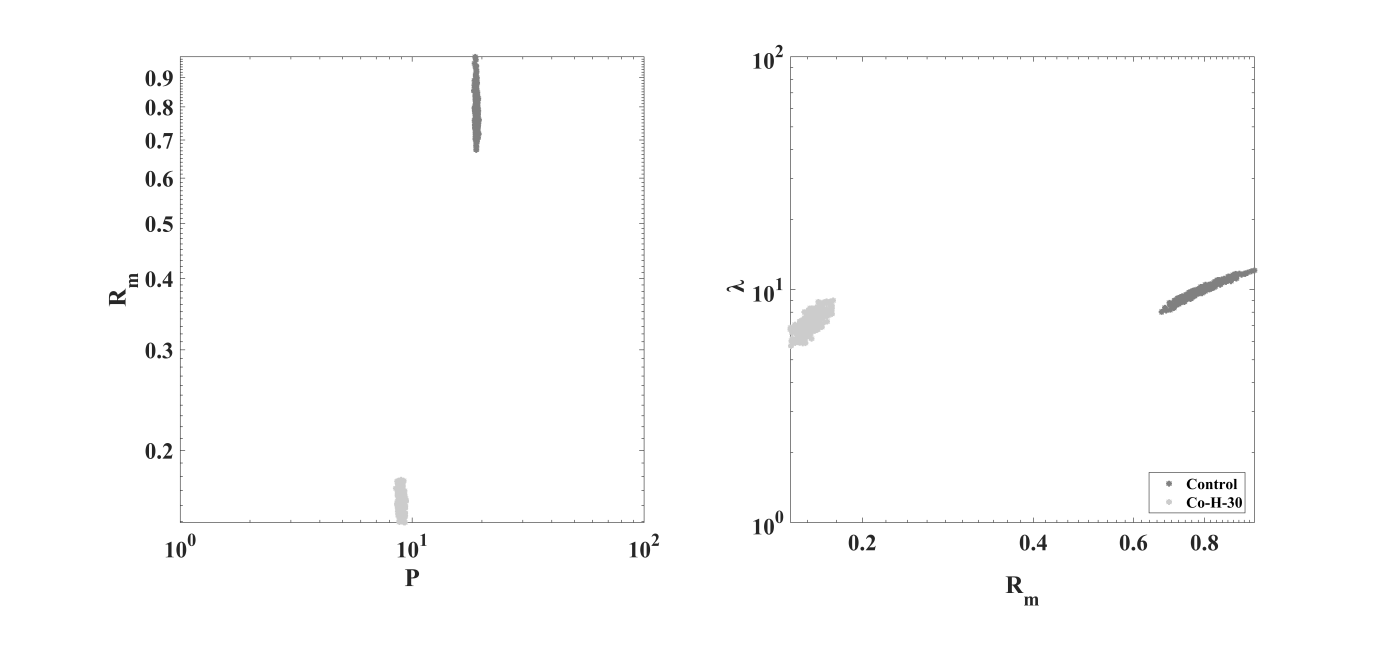
**

**Figure S1 –** Example of the output from Matlab^®^ for the parameter uncertainty regions for the comparison between control and in the presence of 30 mM of Co for hydrogenotrophic conditions.

A

B


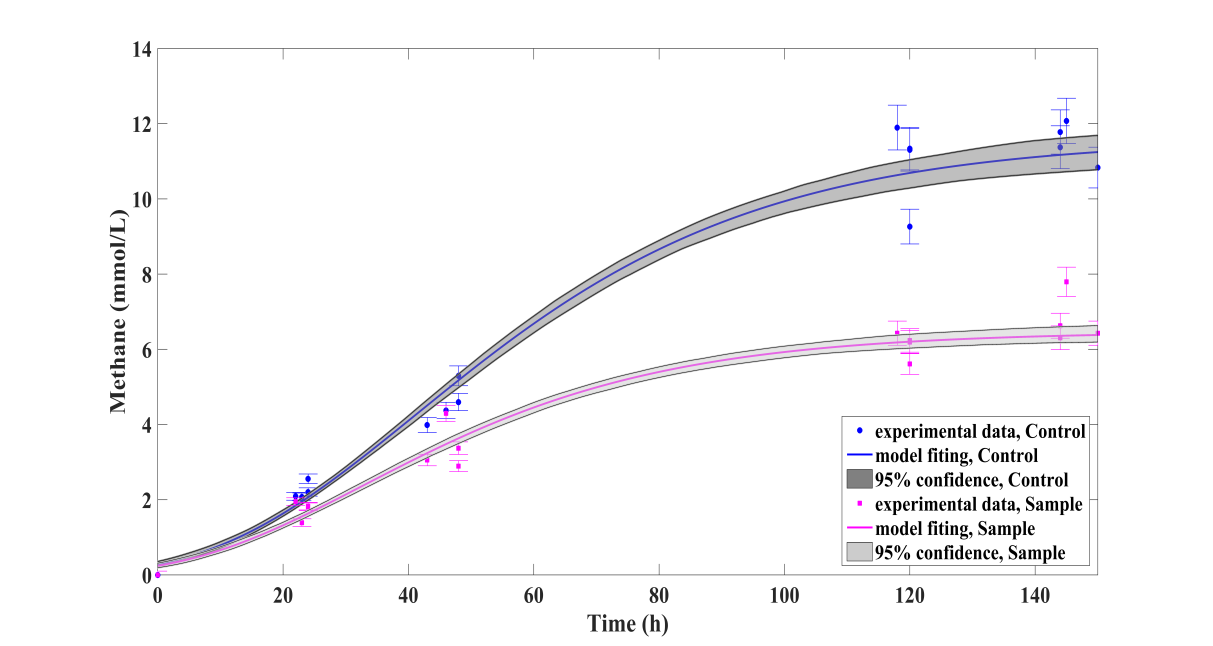


**Figure S2 –** Comparison between a control condition (blue) and a test condition (pink). Points depict experimental data; line represents the fitting model and in grey shadows the respective 95% confidence intervals for each condition. The dashed lines indicate the time points selected for comparison of the different conditions, i.e. 20, 60 and 120 hours.
